# Expression profiles of an inactive aspartic protease (Bla g 2 allergen) in different tissues and developmental stages of the German cockroach (*Blattella germanica*)

**DOI:** 10.1002/arch.21918

**Published:** 2022-06-01

**Authors:** Aaron R. Rodriques, Aaron J. Myers, Michael E. Scharf, Uma K. Aryal, Gary W. Bennett, Ameya D. Gondhalekar

**Affiliations:** ^1^ Department of Entomology Purdue University West Lafayette Indiana USA; ^2^ Entomology and Nematology Department University of Florida Gainesville Florida USA; ^3^ Department of Comparative Pathobiology Purdue University West Lafayette Indiana USA; ^4^ Purdue Proteomics Facility, Bindley Bioscience Center Purdue University West Lafayette Indiana USA

**Keywords:** gene expression, immunoblotting, isoforms, mass spectrometry, proteomics

## Abstract

Tergal glands are found in many insect species and contain constituents such as pheromones, sugars, proteins, and so forth. Preliminary studies have revealed that tergal gland secretions in the German cockroach (*Blattella germanica* L.) contain the human allergen Bla g 2 (*B. germanica* allergen 2), an inactive aspartic protease. Although Bla g 2 protein expression has been detected previously in various German cockroach body parts, including male tergal glands, studies that link protein expression in various life stages and tissues with mRNA and protein abundance have not been conducted. Therefore, the goal of this study was to measure the relative abundances of Bla g 2 protein and mRNA in different tissues and life stages of *B. germanica* using immunoblotting, quantitative PCR, and liquid chromatography‐tandem mass spectrometry (LC‐MS/MS)‐based quantitative profiling. We found that Bla g 2 protein was detected in every sampled tissue, including the male tergal glands. Protein abundance was relatively high in adult males and their tergal glands in comparison to nymphs and virgin females. Similarly, *Bla g 2* mRNA transcript levels were also comparatively higher in male tergal glands and adult males. In conclusion, this study provides new information on the relative abundance and distribution of Bla g 2 allergen, a medically significant protein, in different tissues and developmental stages of the German cockroach and lays the foundation for future studies that aim to determine the function of this protein in *B. germanica* development.

## Highlights

1. *Bla g 2* mRNA was detected in German cockroach tergal glands.
2. Bla g 2 protein and mRNA levels were relatively lower in nymphs as compared to males and gravid females.
3. In comparison to other tissues, Bla g 2 expression was higher in tergal glands.

## Graphical abstract

The mRNA of *Bla g 2* (an inactive aspartic protease) was found to be expressed in situ in the tergal glands of German cockroach adult males. The nymphal stage showed relatively low levels of Bla g 2 expression in comparison to any other life stage. While both Bla g 2 protein and mRNA expression levels were higher in certain body tissues of virgin females, gravid females, and adult males, their abundance was consistently higher in the male tergal glands.

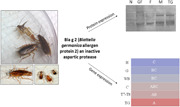

## 1 INTRODUCTION

Tergal glands are structures found on the abdominal segments of various insects, including honeybees, bumblebees, and cockroaches (Dornhaus & Chittka, 2004; Nojima et al., 1999a; Okosun et al., 2015). Honeybee queens secrete tergal gland pheromones that regulate ovarian activation in workers, and bumblebee workers secrete pheromones from their tergal glands that stimulate foraging activity in other workers (Dornhaus & Chittka, 2004; Okosun et al., 2015). Because hymenopteran tergal glands regulate the reproductive hierarchies and resource allocation of bees, they are integral to agricultural and environmental roles played by bees, including honey production and pollination (Wossler & Crewe, 1999). In the urban pest known as the German cockroach (*Blattella germanica*), the adult male possesses tergal glands which produce secretions containing sugars such as maltose, maltotriose, and maltotetraose, as well as other long‐chain carbohydrates (Nojima et al., 1999a). These secretions are thought to act as nuptial gifts for the adult female since the female consumes secretions from the tergal glands while the male attempts to mate with her (Nojima et al., 1999b, 2002). The two tergal glands of *B. germanica* are located on the 7th and 8th tergites of the abdomen (Nojima et al., 2002; Saltzmann et al., 2006). These paired glands are not only utilized during the mating process, as conspecific adult males, gravid females, and nymphs also feed on tergal secretions outside of the context of courtship (Nojima et al., 1999b).

In biochemical analyses of *B. germanica* tergal gland secretions, four major proteins (23, 35–45, 63, and 94 kDa) were visible on SDS‐PAGE gels stained with Coomassie blue (Saltzmann et al., 2006). The 63 kDa protein was identified to be an alpha‐amylase that was then named BGTG‐1 (Saltzmann et al., 2006). Subsequently, Matrix Assisted Laser Desorption Ionization (MALDI)‐based peptide sequencing with individual tergal protein bands, the 63 kDa band, and the large band in the 35–45 kDa region were identified as BGTG‐1 and *B. germanica* allergen 2 or Bla g 2, respectively (Myers, 2015; Myers et al., 2018). While BGTG‐1 is likely involved in the breakdown of long‐chain carbohydrates such as *O–α*‐d‐glucopyranosyl‐(1 → 4)‐*α*‐d‐glucopyranosyl *α*‐d‐glucopyranoside into phagostimulatory sugars (Myers et al., 2018; Nojima et al., 1999a; Saltzmann et al., 2006), the function of Bla g 2 (an inactive aspartic protease) in the tergal glands and cockroach physiology, in general, is not known (Pomés et al., 2002). Bla g 2 has a protein homolog named Per a 2 in the American cockroach *Periplaneta americana*, and just like Bla g 2, Per a 2 is also an inactive aspartic protease of unknown function (Lee et al., 2016; Woodfolk et al., 2016). Other inactive proteases in insects include Lma‐p54, an epicuticular surface protein found in the cockroach *Leucophaea maderae* that may have a binding function (Cornette et al., 2002). In parasitic arthropods such as ticks, mites, and mosquitoes, inactive proteases or pseudoproteases are proposed to play important roles in egg formation, hemoglobin transport, immunomodulation, regulation, and acceleration of fibrin formation (Fernando & Fischer, 2020).

Bla g 2 is also one of the many allergen proteins produced by *B. germanica* and can be detected in their feces, exoskeletons, and egg casings (Arruda et al., 1995; Pomés et al., 2002). Bla g 2 and other allergens produced by *B. germanica* cause allergies, asthma, and other ailments of the immune and respiratory system (Arruda, 2005), and thus are a threat to human health. Bla g 2 is a potent allergen that is ubiquitous in household settings, especially inner‐city housing (Gore & Schal, 2007; Wang & Bennett, 2009). Arruda et al. (1995) determined the relative expression of the Bla g 2 protein in several *B. germanica* tissues, including the esophagus, crop, proventriculus, gut, legs, wings, egg casings, fat bodies, salivary glands, and trachea. Although Bla g 2 protein was detected in the secretions of the tergal glands (Myers, 2015), and Bla g 2 protein expression occurs in multiple tissues (Arruda et al., 1995), certain variables related to Bla g 2 expression have yet to be investigated. For example, Bla g 2 protein expression in the tergal glands relative to other tissues of the German cockroach has not been determined. Similarly, the relative expression of *Bla g 2* mRNA in the cockroach tergal glands and other tissues, as well as in different cockroach life stages and sexes, remains unknown. The large, detectable quantities of Bla g 2 protein present in the German cockroach living environment can potentially contaminate body parts such as wings (Arruda et al., 1995) and the same could be true for the presence of Bla g 2 protein in tergal secretions. Alternatively, Bla g 2 could be produced primarily by other body tissues (e.g., alimentary canal) and then transported to wings and tergal glands. Additionally, the male wing raising before mating (Nojima et al., 1999b) may be a behavior that has evolved to make Bla g 2 more readily available, rather than a behavior to enable tergal feeding. As such, it is important to determine if the Bla g 2 protein detected in the tergal glands is actually translated in these glands from mRNA. In another cockroach species (*L. maderae*) mRNA of the cuticular inactive aspartic protease protein (Lma‐p54) is detectable in the abdominal tergal glands (Cornette et al., 2002). Therefore, we hypothesize that *Bla g 2* mRNA and protein are expressed in multiple tissues, with relatively high expression in the male tergal glands. To test this hypothesis, the objectives of this study were to (i) determine the relative expression of *Bla g 2* mRNA and (ii) quantify the expression of Bla g 2 protein in different tissues and life stages of the German cockroach.

## 2 MATERIALS AND METHODS

### 2.1 Insect rearing

All cockroaches were reared in a Percival Scientific™ environmental chamber at 25.5°C temperature, 50% relative humidity, and a 12:12 (L:D) photoperiod. The cockroaches of the Johnson Wax insecticide susceptible (JWax‐S) strain, which were maintained in plastic boxes and raised on rodent chow (Harlan‐ Teklad #8604) were used for all experiments. More detailed information on the JWax‐S cockroach strain and its rearing can be found in Fardisi et al. (2017).

### 2.2 Life stages used for gene and protein expression analysis

Four German cockroach life stages were used in this study; nymphs, virgin males (males), virgin females (females), and gravid females carrying an ootheca. All life stages were individually held in vented 1 oz. portion cups for 3−7 days before their use for RNA and protein extraction. Portion cups were provisioned with rodent chow and a water source. The nymphs used were at their late 3rd or early 4th instar stage. Freshly molted males and females aged for 7 days past their imaginal molt were used. Gravid females with fully extended and developed oothecae were used for gene and protein expression experiments.

### 2.3 Dissection and processing of tissues for gene and protein expression analysis

Dissections were performed under a Nikon SMZ‐10 stereo microscope (Nikon) in wax‐poured 35 × 15 mm Petri dishes (Fisher Scientific). Before RNA isolation, the tissues were dissected and separated into 1.5 ml Eppendorf tubes containing 250 μl of RNA*later* stabilization solution (Thermo Fisher Scientific), and held at 4°C for 24 h, after which the RNA*later* solution was decanted following the manufacturer protocol. Subsequently, the tissues were stored at −80°C until RNA isolation. The whole‐body samples of respective life stages were cut into ca. 0.25–0.50 cm‐sized pieces with dissection scissors and then processed in RNA*later* using the same protocol described above for various tissues. To isolate protein from whole bodies or tissues, homogenization was conducted in PBS (pH 7.4 and 0.1 M) using a glass mortar and Teflon pestle. Homogenates were then centrifuged for 15 min at 15,000*g* and 4°C to obtain nuclear and mitochondrial fraction pellets and supernatant containing the cytosolic protein fraction. Only the cytosolic protein fractions were used for further analyses.

### 2.4 RT‐qPCR

RNA isolation was done using the SV Total RNA Isolation System (Promega), according to the manufacturer's instructions. Next, RNA was reverse‐transcribed to cDNA using the iScript® cDNA synthesis kit (Bio‐Rad). Each 20 µl cDNA synthesis reaction consisted of 4 µl of reaction buffer, 1 µl reverse transcriptase, 50 µg of total RNA (variable volume up to 15 µl), and nuclease‐free water to make up the volume. Reactions for cDNA synthesis were incubated in a thermal cycler at 25°C for 5 min, 42°C for 30 min, and 85°C for 5 min. Each cDNA sample was then split into two 10 µl aliquots and stored at −20°C until conducting RT‐qPCR. To determine *Bla g 2* mRNA expression levels, RT‐qPCR (real‐time‐quantitative polymerase chain reaction) experiments were conducted following the protocol outlined by Myers et al. (2018).

To enable the calculation of reference gene expression or critical threshold values, all cDNA samples were spiked with *pGEM*™ plasmid DNA (50 ng) and M13 primers were used to amplify a 200 bp “M13” region of the plasmid (Myers et al., 2018). Primer sequences for the target gene (*Bla g 2*) and reference gene (M13 region of *pGEM*™ plasmid) are shown in Table 1. Each RT‐qPCR reaction included the following: SYBR® SensiMix at 1× concentration (Bioline), forward and reverse primers (final concentration 0.5 µM), 1 μl cDNA template (prepared from 50 µg of total RNA), and Nanopure water to make up the volume. Thermal cycler conditions used for RT‐qPCR were identical to those used by Myers et al. (2018). RT‐qPCR experiments were conducted with three biological replicates for each life stage (i.e., whole‐body samples) and tissues. Each biological replicate consisted of 10 cockroaches. For every biological replicate, three technical replicates were conducted. The 2^−ddCT^ method of quantifying gene expression was used (Livak & Schmittgen, 2001). In brief, for any particular life stage or tissue, the critical threshold (CT) values generated during RT‐qPCR were converted into dCT values by subtraction of the average CT value for the noncoding region of the pGEM plasmid that was amplified by the universal M13 primers. Any tissue that would not yield CT values in all three biological replicates was excluded from further analysis. The dCT values were then converted into −ddCT values by subtraction of the average head dCT value of that respective life stage. These −ddCT values were then exponentialized to 2^−ddCT^ to obtain relative differences or fold change in *Bla g 2* mRNA expression between different samples. For statistical analysis, the data for each life stage and their tissues were analyzed individually using ANOVA and Student's *t* test in SAS‐JMP Pro 15.

**Table 1:** Primer sequences used for the target (*Bla g 2*) and reference (M13 region of the *pGEM*™ plasmid) genes

Gene/Primer ID	Forward primer (5' to 3')	Reverse primer (5' to 3')
Bla g 2 (GenBank accession no. AAA86744.1)	GCCCTAATGCACTGAAAGGA	TCCAATCAGAACCTCCGAAA
M13^a^ (pGEM reference gene primers)	GTAAAACGACGGCCAGT	AACAGCTATGACCATG

^a^ M13 region of the pGEM plasmid was used because, as per a previous study Myers et al., other reference genes, such as beta actin and ribosomal protein L13a, did not show consistent expression when comparing different German cockroach tissues (Myers et al., 2018).

### 2.5 Synthesis of antigens and polyclonal antibodies for Western blots

The primary polyclonal mouse antibody for Bla g 2 was obtained from GenScript®. This antibody binds to an epitope of the sequence DTGSAVGRGIEDSLC. The antibody provided by GenScript® was >99% pure.

### 2.6 Determination of Bla g 2 protein expression profiles

The protein content of all cockroach life stages and tissues was determined using the Bradford assay with bovine serum albumin as a standard (Bradford, 1976). Next, cytosolic protein fractions (40 μg per life stage or tissue) were diluted 1:1 using 2× Laemmli SDS‐PAGE sample buffer (Bio‐Rad) containing beta mercaptoethanol and heated for 10 min at 95°C. The diluted and denatured protein fractions (40 μg concentration) were loaded in individual lanes/wells of a 10% SDS‐PAGE polyacrylamide gel (acrylamide:bis, 30:0.8). Proteins were separated based on their molecular weight by subjecting the gel to 200 V for 15 min and then to 150 V for 45 min using the Criterion™ Cell (Bio‐Rad). The SDS‐PAGE‐separated proteins were transferred onto nitrocellulose membranes using the Criterion™ Blotter (Bio‐Rad). After electrophoretic transfer, the membranes were blocked by incubation for 1 h in TBS buffer containing nonfat dry milk at a concentration of 10 mg/ml (Bio‐Rad). The membranes were then incubated for 1 h at room temperature in Bla g 2 mouse polyclonal antibody diluted 1:4000 in TBS + nonfat dry milk.

Membranes were washed twice for 10 min each with TBS‐Tween/Triton buffer, once with TBS buffer for 10 min, and then incubated for 1 h at room temperature with goat anti‐mouse AP‐conjugated secondary antibody diluted 1:4000 in TBS + nonfat dry milk. The membranes were then washed four times with TBS‐Tween/Triton buffer and developed using SIGMAFAST™ BCIP®/NBT alkaline phosphatase (Millipore‐Sigma) as described in the manufacturer's protocol. The blots were imaged using the Molecular Imager® ChemiDoc™ XRS^+^ Imaging System (Bio‐Rad). Band intensities were quantified from three replicate blots per experiment using ImageJ™ (NIH). Intensities were normalized within blots by subtraction of a band intensity control value. The control was a randomly selected area within the nitrocellulose membrane that did not contain bands. The size of the control band area used for intensity measurement was the same as the respective protein band area. The normalized intensities were then averaged across replicates. Statistical analysis consisted of ANOVA and Student's *t* test.

### 2.7 Peptide sequencing by liquid chromatography‐tandem mass spectrometry (LC‐MS/MS)

To obtain secretions from male tergal glands, sterile filter paper strips were placed in glandular reservoirs of adult males for 5−10 s (Myers et al., 2018; Saltzmann et al., 2006). After that the paper strips were submerged in PBS (pH 7.4) for 10−15 min to allow protein dissipation into the buffer and then discarded (Myers et al., 2018; Saltzmann et al., 2006). The supernatant with dissolved proteins was used as a source of secreted tergal proteins (Myers et al., 2018; Saltzmann et al., 2006). To obtain proteins from the dissected male tergal gland tissue and gravid female whole bodies, they were homogenized in PBS (pH 7.4) and subsequently subjected to differential centrifugation as described in Section 2.2. The tergal gland secretion, tergal gland tissue, and gravid female protein samples (cytosolic fractions) were separated using 1D SDS‐PAGE gel as described in Section 2.6. The gels were stained with Coomassie Gelcode Blue Stain Reagent for 1 h (Thermo Scientific #24590). After destaining, gel bands of interest were excised and subjected to in‐gel tryptic digestion and LC‐MS/MS analysis at the Purdue Proteomics Facility as described previously (Hedrick et al., 2015). In‐gel digested peptides were extracted using 60% ACN/5% trifluoroacetic acid (TFA) and dried in a vacuum centrifuge to prepare for LC‐MS/MS analysis.

Peptides were resuspended in 10μl of 3% ACN/0.1% Formic Acid (FA)/96.9% MilliQ, and 5 μl was used for LC‐MS/MS analysis Peptides were analyzed in the Dionex UltiMate 3000 RSLC nano System coupled to the Q‐Exactive High‐Field (HF) Hybrid Quadrupole Orbitrap MS (Thermo Fisher Scientific) as described elsewhere (Connelly et al., 2018; Mohallem & Aryal, 2020; Zembroski et al., 2021) using 130‐min LC method. The MS data were acquired with a Top 20 data‐dependent MS/MS scan method with a maximum injection time of 100 ms, a resolution of 120,000 at 200 *m/z*. Fragmentation of precursor ions was performed by high‐energy C‐trap dissociation (HCD) with the normalized collision energy of 27 eV. MS/MS scans were acquired at a resolution of 15,000 at *m/z* 200. The dynamic exclusion was set at 20 s to avoid repeated scanning of identical peptides.

### 2.8 MS/MS data analysis and bioinformatics

The raw LC‐MS/MS data were processed using MaxQuant (v1.6.3.3) (Cox & Mann, 2008), with the spectra matched against the *B. germanica* protein database downloaded from Uniprot (http://www.uniprot.org) on 05/20/2018. Data were searched using trypsin/P and LysC enzyme digestion allowing for up to two missed cleavages. MaxQuant search was set to 1% FDR both at the peptide and protein levels. The minimum peptide length required for database search was set to seven amino acids. Precursor mass tolerance of ±10 ppm, MS/MS fragment ions tolerance of ±20 ppm, and oxidation of methionine protein N‐terminal acetylation (K) were set as the variable modifications and carbamidomethylation of cysteine (C) was set as a fixed modification. The “unique plus razor peptides” were used for peptide quantitation. Razor peptides are the nonredundant, nonunique peptides assigned to the protein group with most other peptides. MS/MS counts were used for relative protein abundance determination. Proteins detected with at least one unique peptide and at least two MS/MS counts were used for relative quantitation.

## 3 RESULTS

### 3.1 mRNA expression profile

**Figure 1:**
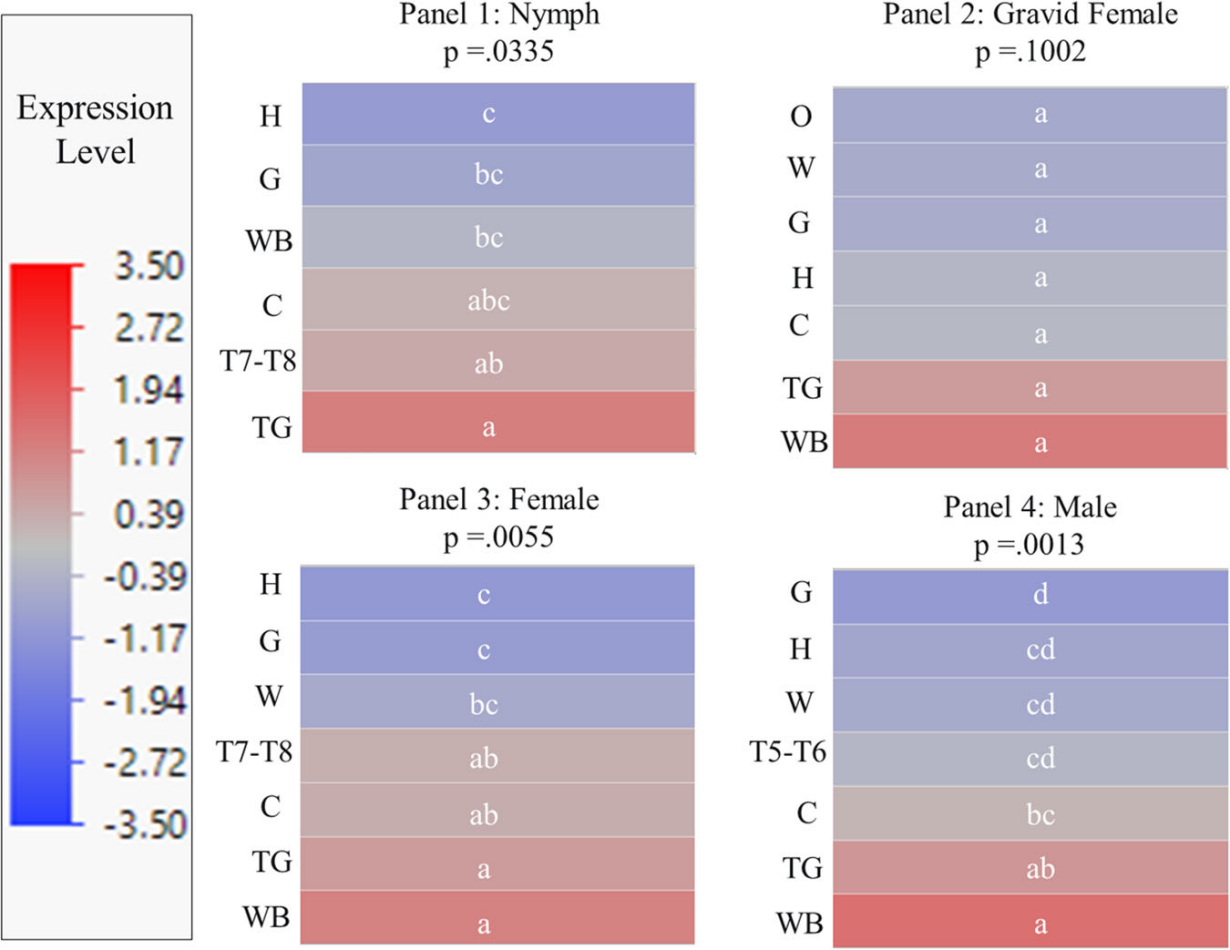
Heat maps generated using *z* scores (derived from −ddCT values) that show *Bla g 2* mRNA expression levels for different life stages and body tissues of German cockroaches. The red−blue color spectrum indicates the range of *Bla g 2* mRNA expression; tissues associated with a red coloration had relatively high expression of *Bla g 2*, and tissues associated with a blue coloration had relatively low expression of *Bla g 2*. The white connecting letters (a, b, ab, c, abc, bc, d, cd) indicate if a statistically significant difference was found among various whole body and tissue samples. Within each heat map, samples or tissues with different connecting letters were statistically significant (*p* < 0.05; Student's *t* test). Panel 1 shows a heat map for the nymphal head (H), nymphal gut (G), nymphal 7−8th tergites (T7−T8), nymphal carcass (C), nymphal whole body (WB), and male tergal glands (TG). Panel 2 shows a heat map for the gravid female head, gravid female wings (W), gravid female gut, gravid female carcass, gravid female ootheca (O), gravid female whole body, and male tergal glands. Panel 3 shows a heat map for the female head, female wings, female gut, female 7−8th tergites, female carcass, female whole body, and male tergal glands. Panel 4 shows a heat map for the male head, male wings, male gut, male 5−6th tergites (T5−T6), male carcass, male whole body, and male tergal glands.

For all life stages, the head tissue exhibited the lowest levels of *Bla g 2* mRNA expression and hence it was used as a baseline tissue for gene expression analysis (Figure 1). Male tergal glands showed the highest *Bla g 2* expression level in comparison to whole bodies and tissues of the nymphal stage (Figure 1, Panel 1). *Bla g 2* mRNA levels in the male tergal glands were significantly higher relative to nymphal head, gut, and whole‐body expression (*p* < 0.05). The 7−8th nymphal tergites showed higher expression than the head (*p* = 0.0396) but were not statistically different from the other tissues.

Although *Bla g 2* transcript levels in the whole body samples of gravid females were at higher levels in comparison to the head, wing, gut, and carcass tissues, these differences were not statistically supported (*p* = 0.1002; Figure 1, Panel 2). Similarly, male tergal glands, which showed the second highest *Bla g 2* expression after the whole body samples, did not exhibit a statistical difference compared to any other tissues.

In the analysis of nongravid or virgin females, both the 7−8th tergites and the carcass showed higher *Bla g 2* expression than the head and gut tissues (*p* < 0.05; Figure 1, Panel 3). Both the female whole body and male tergal glands showed higher *Bla g 2* expression than the head, wing, and gut tissues (*p* < 0.05). No difference was observed between the whole body and tergal gland tissues.

The male carcass showed higher expression than the gut but did not show statistically higher expression compared to any other tissues (*p* < 0.05; Figure 1, Panel 4). The male tergal glands showed higher expression than the head, gut, wings, and 5−6th tergites. The whole body also showed higher expression than the head, gut, wings, 5−6th tergites, and carcass (*p* < 0.05). Overall, the male whole body and tergal glands showed the highest levels of *Bla g 2* expression.

### 3.2 Bla g 2 protein expression analysis using immunoblotting and LC‐MS/MS

The polyclonal antibody predominantly detected two Bla g 2 bands of ca. 35 and 70 kDa in size. The 35 kDa band was close to the predicted molecular weight of different Bla g 2 isoforms (29–36 kDa), as reported in Table 2. The 35 and 70 kDa bands were present in the whole‐body and tissue samples of all cockroach life stages (Panel 1 of Figures 3, 4, 5, 6, 7). Another high mass (~95–105 kDa) Bla g 2 band was also detectable in the gravid female whole‐body sample (Panel 1 of Figures 3 and 5). Throughout the remainder of this paper, the high mass band is referred to as the 105 kDa band.

**Table 2:** Bla g 2 isoforms detected in the 35 and 70 kDa bands of the male tergal secretion and the 105 kDa band detected in the gravid female whole body sample

Protein ID^a^	Molecular weight^a^	Location
gi|1370647966	29.613	Band 1, Band 2, 105 kDa band
gi|1370635791	34.072	Band 1, Band 2, 105 kDa band
gi|62738637	36.032	Band 1, Band 2

^a^ All protein IDs and molecular weights are from GenBank.

**Figure 2:**
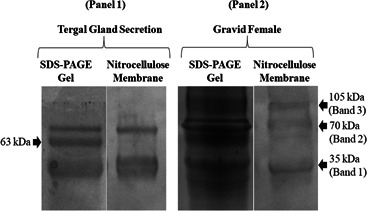
(Panel 1) An SDS‐PAGE gel and a Western blot nitrocellulose membrane showing Bla g 2 protein bands from the male tergal gland secretion. (Panel 2) An SDS‐PAGE gel and a Western blot nitrocellulose membrane showing protein bands from a gravid female cytosolic protein fraction. The gel band between the 35 kDa gel band and the 70 kDa gel band in Panel 1 or the tergal gland secretion SDS‐PAGE gel that did not produce a distinct band on the Western blot nitrocellulose membrane is a BGTG‐1 or alpha‐amylase protein band (63 kDa; GenBank accession no. AY945930; Saltzmann et al., 2006).

The 35, 70, and 105 kDa protein bands detected on the Western blot nitrocellulose membranes were also visible on the 1D‐SDS‐PAGE gel after Coomassie staining (Figure 2). Performing LC‐MS on the 35 and 70 kDa protein gel bands from males and the 105 kDa protein gel band of the gravid female resulted in the detection of peptide sequences belonging to different Bla g 2 isoforms in all three of these bands (Tables 2 and 3). These sequencing results confirmed the Western blot results that all three bands represented Bla g 2 peptides. The 35 kDa band contained 56 proteins, and the overall relative abundance of Bla g 2 isoforms in this band was 34.14%. The 70 kDa band contained 153 proteins, and Bla g 2 protein abundance in this band was 42.32%. Lastly, the 105 kDa band contained a much higher number of proteins (241) with Bla g 2 peptide abundance of 0.083% (Table 3). These results suggest that majority of Bla g 2 protein may exist as dimer endogenously.

**Table 3:** Data from LC‐MS peptide sequencing showing the total number of proteins, percent relative abundance of Bla g 2 protein, and peptides mapped to Bla g 2 in the two gel bands of male tergal secretions and the 105 kDa band from the gravid female whole body sample

Bands	Total number of proteins	% Relative abundance of Bla g 2 protein^a^	Peptides mapped to Bla g 2
Band 1	56	34.14	AYVNPINEAIGCVVER VSSLPDVTFVISGK AVEVPMSIDELTSPK AEEVTFFDTGR LDGVKIGDTTVAPAGTQAIIDTSK IPSLPDVTFVINGR FRLDGVK TVLENFVEENLIAPVFSIHHAR FQDGEHFGEIIFGGSDWK YVDGEFTYVPLVGDDSWK YISDGNVQVKFFDTGSAVGR IGDTTVAPAGTQAIIDTSKAIIVGPK AYVNPINEAIGCVVEK YISDGNVQVK IGDTTVAPAGTQAIIDTSK FFDTGSAVGR LVHVFINTQYAGITK
Band 2	153	42.32	AYVNPINEAIGCVVER VSSLPDVTFVISGK MIGVVESRD MIGVVESR VFINTQYAAK SIIVGPEAQISNINK AVEVPMSIDELTSPK AEEVTFFDTGR LDGVKIGDTTVAPAGTQAIIDTSK IPSLPDVTFVINGR FRLDGVK TVLENFVEENLIAPVFSIHHAR FQDGEHFGEIIFGGSDWK YVDGEFTYVPLVGDDSWK AYVNPINEAIGCVVEKTTTR YISDGNVQVKFFDTGSAVGR IGDTTVAPAGTQAIIDTSKAIIVGPK AYVNPINEAIGCVVEK YISDGNVQVK IGDTTVAPAGTQAIIDTSK FFDTGSAVGR LVHVFINTQYAGITK
Band 3	241	0.083	AVEVPMSIDELTSPK VSSLPDVTFVISGK AEEVTFFDTGR SIIVGPEAQISNINK

^a^ % Relative abundances are derived from the ratios of the sum of Bla g 2 MS counts over the sum of MS counts of all proteins.

**Figure 3:**
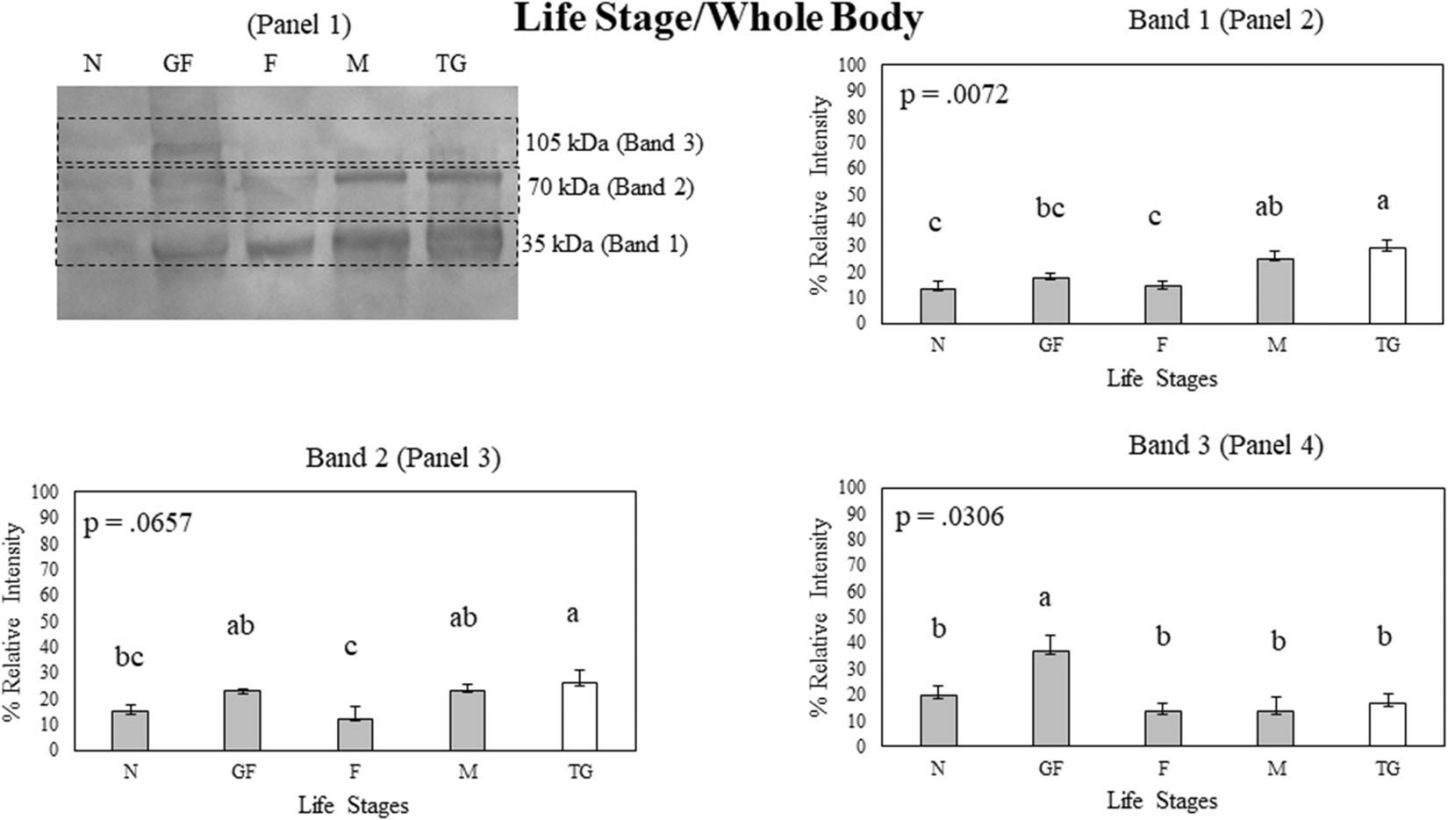
(Panel 1) A Western blot showing Bla g 2 protein expression in whole body samples of nymphs (N), gravid females (GF), females (F), males (M), and male tergal glands (TG) on a nitrocellulose membrane. Bla g 2 exhibits multiple bands in a Western blot, with Band 1 (Panel 2) having the lowest molecular weight, Band 2 (Panel 3) having an intermediate molecular weight, and Band 3 (Panel 4) having the highest molecular weight. Protein expression data were compared between whole body and tergal gland samples using Student's *t* test. Error bars indicate standard error values. Within each panel that shows relative band intensities, life stages or tissue samples connected with the different letters exhibit statistically significant differences in gene expression (*p* < 0.05).

Statistical analysis of Bla g 2 band intensity data from Western blots among different life stages was conducted (Figure 3, Panels 2‒4). In Band 1, Bla g 2 expression in males was higher as compared with nymphs and females (*p* < 0.05), but it was statistically similar to gravid females (*p* > 0.05) (Figure 3, Panel 2). For the same band, Bla g 2 expression in the male tergal gland tissue was similar to expression levels in the adult male whole body; however, it was higher than the expression in nymphs, gravid females, and females. In Band 2, both the gravid female and male whole‐body samples had higher Bla g 2 expression than the females and nymphs. Bla g 2 expression in the male tergal gland tissue was also higher than in the nymph and female whole bodies (Figure 3, Panel 3). In Band 3, gravid females had higher Bla g 2 expression than nymphs, females, males, and male tergal glands (Figure 3, Panel 3). This is because this high mass band 3 (~95–105 kDa) was mostly undetectable in life stages other than the gravid female.

**Figure 4:**
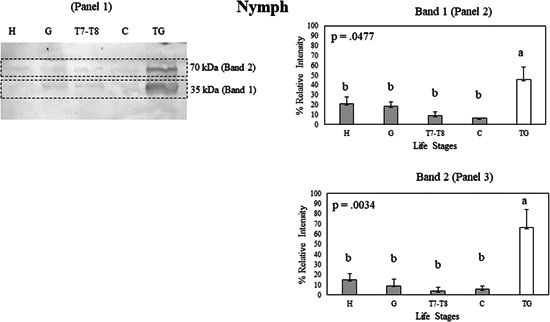
(Panel 1) A Western blot showing Bla g 2 protein expression in tissue samples of the nymphal head (H), nymphal gut (G), nymphal 7−8th tergites (T7−T8), nymphal carcass (C), and male tergal glands (TG) on a nitrocellulose membrane. Bla g 2 exhibits multiple bands in a Western blot, with Band 1 (Panel 2) having the lowest molecular weight, and Band 2 (Panel 3) having the highest molecular weight. Protein expression data were compared among tissue samples using Student's *t* test. Error bars indicate standard error values. Within each panel that shows relative band intensities, life stages or tissue samples connected with the different letters exhibit statistically significant differences in gene expression (*p* < 0.05).

**Figure 5:**
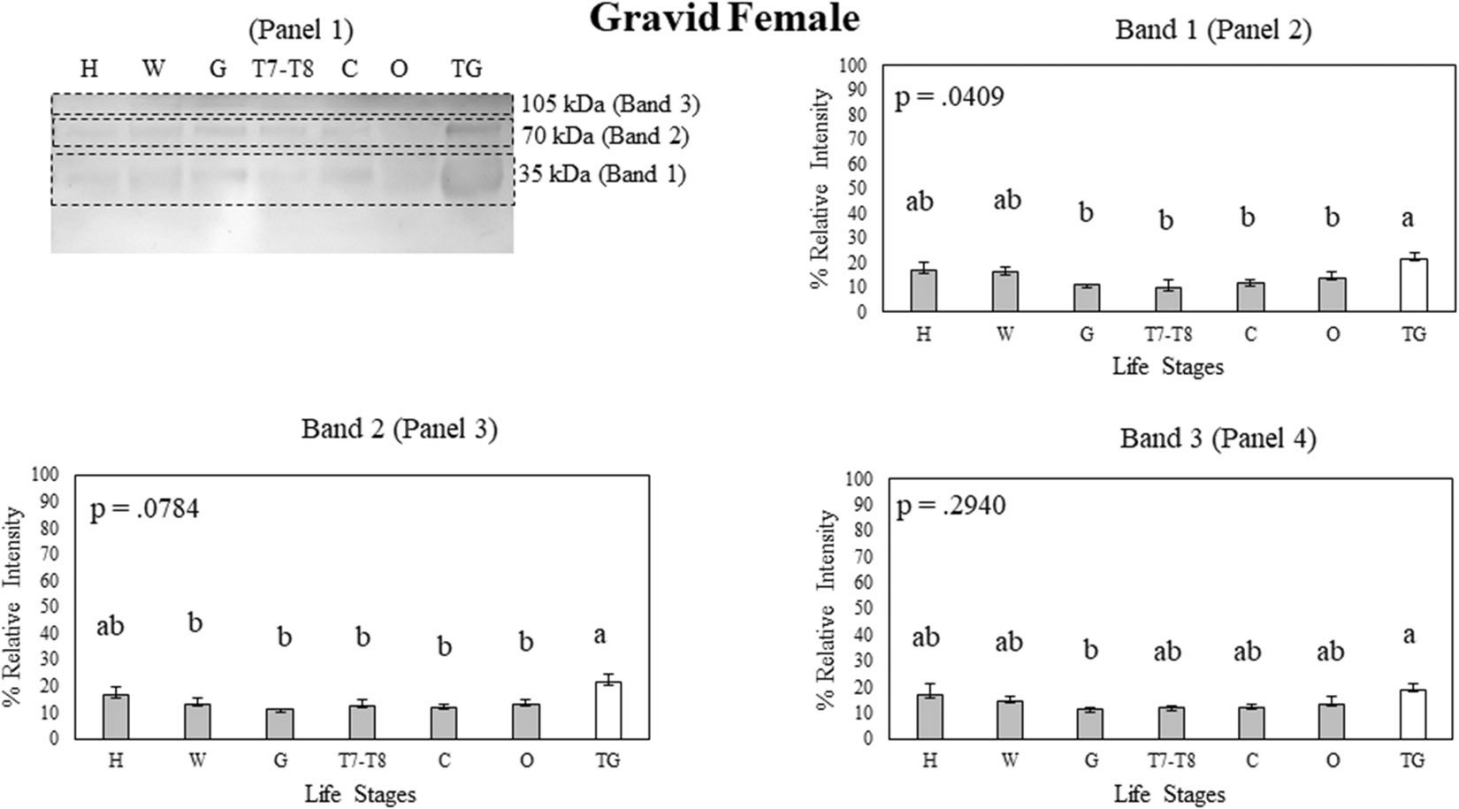
(Panel 1) A Western blot showing Bla g 2 protein expression in tissue samples of the gravid female head (H), gravid female wings (W), gravid female gut (G), gravid female 7−8th tergites (T7−T8), gravid female carcass (C), gravid female ootheca (O), and male tergal glands (TG) on a nitrocellulose membrane. Bla g 2 exhibits multiple bands in a Western blot, with Band 1 (Panel 2) having the lowest molecular weight, Band 2 (Panel 3) having an intermediate molecular weight, and Band 3 (Panel 4) having the highest molecular weight. Protein expression data were compared between whole body and tissue samples of each life stage using Student's *t* test. Error bars indicate standard error values. Within each panel that shows relative band intensities, life stages or tissue samples connected with the different letters exhibit statistically significant differences in gene expression (*p* < 0.05).

**Figure 6:**
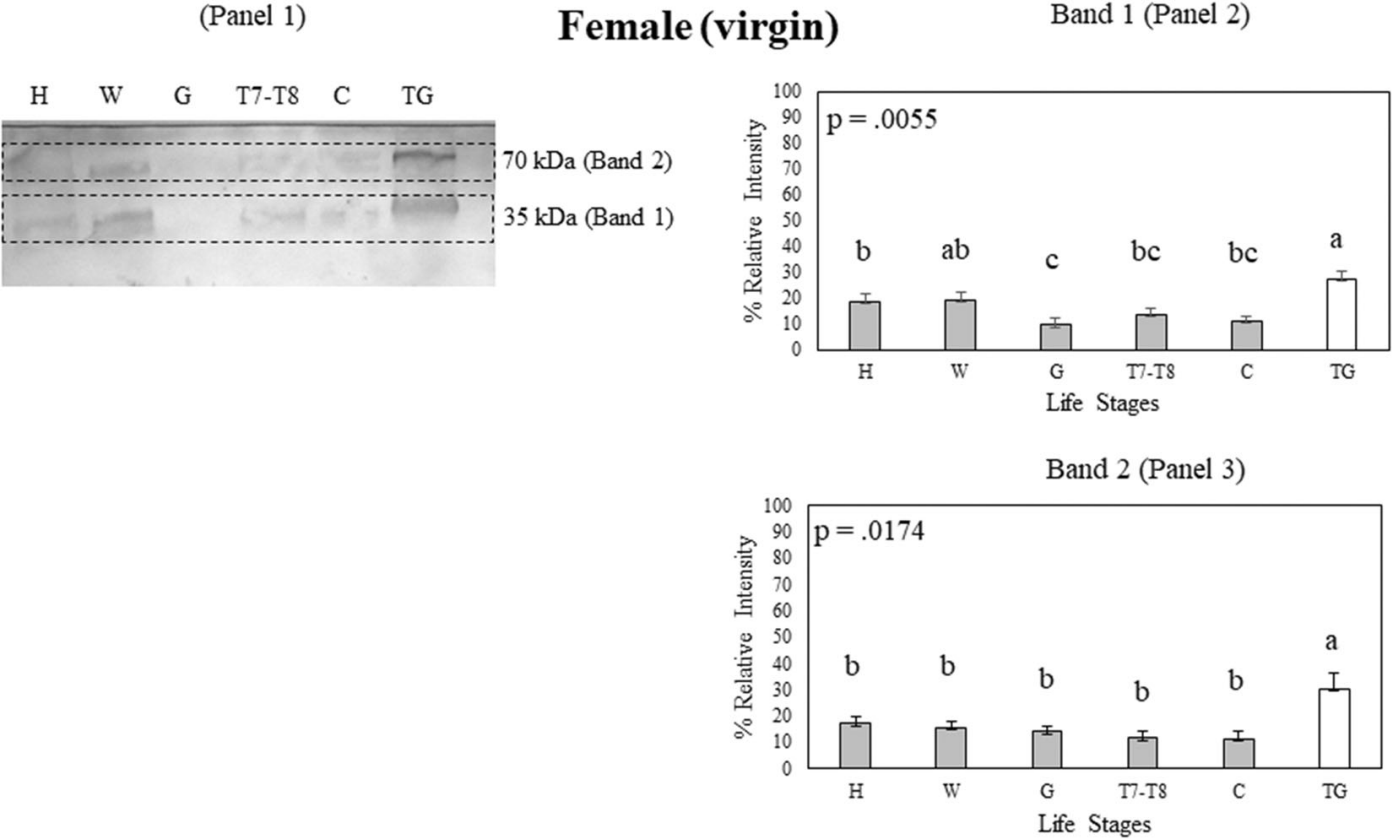
(Panel 1) A Western blot showing Bla g 2 protein expression in tissue samples of the female head (H), female wings (W), female gut (G), female 7−8th tergites (T7−T8), female carcass (C), and male tergal glands (TG) on a nitrocellulose membrane. Bla g 2 exhibits multiple bands in a Western blot, with Band 1 (Panel 2) having the lowest molecular weight and Band 2 (Panel 3) having the highest molecular weight. Protein expression data were compared among tissue samples using Student's *t* test. Error bars indicate standard error values. Within each panel that shows relative band intensities, life stages or tissue samples connected with the different letters exhibit statistically significant differences in gene expression (*p* < 0.05).

**Figure 7:**
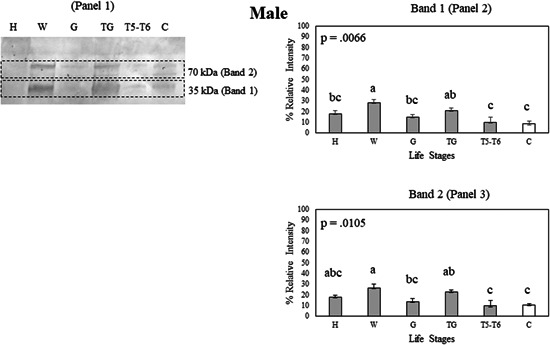
(Panel 1) A Western blot showing Bla g 2 protein expression in tissue samples of the male head (H), male wings (W), male gut (G), male tergal glands (TG), male 5−6th tergites (T5−T6), and male carcass (C) on a nitrocellulose membrane. Protein expression data were compared between whole body and tissue samples of each life stage using Student's *t* test. Bla g 2 exhibits multiple bands in a Western blot, with Band 1 (Panel 2) having the lowest molecular weight and Band 2 (Panel 3) having the highest molecular weight. Protein expression data were compared among tissue samples using Student's *t* test. Error bars indicate standard error values. Within each panel that shows relative band intensities, life stages or tissue samples connected with the different letters exhibit statistically significant differences in gene expression (*p* < 0.05).

Bla g 2, tissue expression analysis of different life stages was also conducted (Figures 4, 5, 6, 7 and Supporting Information: Figure S1). Tissue analysis of nymphs showed that the male tergal glands had higher Bla g 2 expression compared to the nymphal head, gut, carcass, and 7−8th tergites in Band 1 (*p* < 0.05; Figure 4, Panel 2). There was no difference between Bla g 2 expression of the nymphal head, gut, carcass, and 7−8th tergites in Band 1 (*p* > 0.05; Figure 4, Panel 2). In Band 2, the male tergal glands also had higher Bla g 2 content as compared to the nymphal head, gut, carcass, and 7−8th tergites in Band 2 (Figure 4, Panel 3). As with Band 1, there was no difference between the Bla g 2 expression of the nymphal head, gut, carcass, and 7−8th tergites in Band 2.

The analysis of gravid females showed multiple statistical differences between gravid female tissues and the male tergal glands (*p* < 0.05, Figure 5, Panels 2‒4). The male tergal glands showed higher Bla g 2 expression compared to the gravid female gut, carcass, 7−8th tergites, and ootheca in Band 1 (*p* < 0.05, Figure 5, Panel 2). No difference could be found between the male tergal glands and the gravid female wings in Band 1 (*p* > 0.05, Figure 5, Panel 2). The male tergal glands also showed higher Bla g 2 expression compared to the gravid female wings, gut, carcass, 7−8th tergites, and ootheca in Band 2 (*p* < 0.05, Figure 5, Panel 3). The 105 kDa protein band (Band 3), which was unique to gravid females, was detectable at low levels in different body tissues but no tissue‐dependent statistical differences in its expression were observed (*p* > 0.05; Figure 5, Panel 4).

In females, both the head and wing samples exhibited higher Bla g 2 expression than the female gut in Band 1 (*p* < 0.05, Figure 6, Panel 2). The male tergal glands had higher expression than the female head, gut, carcass, and 7−8th tergites in Band 1 (*p* < 0.05; Figure 6, Panel 2). In Band 2, the male tergal glands had higher Bla g 2 expression than the female head, wings, gut, carcass, and 7−8th tergites (Figure 6, Panel 3). As observed with the gravid female tissue samples, the male tergal glands also had the highest expression of Bla g 2 in comparison to nongravid female tissues.

With male tissues, wings had higher Bla g 2 expression compared to the head, gut, 5−6th tergites, and carcass in Band 1 (Figure 7, Panel 2). Male tergal glands also had high expression compared to the 5−6th tergites and carcass in Band 1 (Figure 7, Panel 2). In Band 2, wings had higher Bla g 2 expression compared to the gut, 5−6th tergites, and carcass (Figure 7, Panel 3). Male tergal glands also had high expression compared to the 5−6th tergites and carcass in Band 2 (Figure 7, Panel 3).

## 4 DISCUSSION

These results build upon previous research by Arruda et al. (1995) that quantified Bla g 2 protein content in the gravid female esophagus, crop, proventriculus, gut, legs, wings, egg casings, fat bodies, salivary glands and trachea, as well as other research on Bla g 2 and the German cockroach tergal glands (Arruda, 2005; Arruda et al., 1995; Myers, 2015; Pomés et al., 2002; Saltzmann et al., 2006). We report a series of *Bla g 2* mRNA and protein expression profiles for the whole bodies of nymphs, males, females, and gravid females of *B. germanica*, as well as different body tissues of these life stages, including the head, wings, gut, 5−6th tergites, 7−8th tergites, carcass, and ootheca. While previous analysis used MALDI TOF MS‐based analysis, we applied high‐throughput LC‐MS/MS for identification and relative quantitation of Bla g 2 isoforms. By conducting *Bla g 2* mRNA expression and Western blot protein detection experiments with male‐specific tergal glands, we have shown for the first time that Bla g 2 protein detected in these glands is translated from mRNA in situ, rather than being transferred from the environment or other body tissues.

In comparison to the nymphal stage, the male tergal glands and whole bodies of every other life stage that was tested showed relatively high levels of *Bla g 2* mRNA expression. Similarly, in the Western blot experiments, whole bodies of every life stage had equal or somewhat higher Bla g 2 protein expression than the nymphal stage. Therefore, for the whole body samples, both qPCR and Western blot data were mostly in agreement. Similarly, both qPCR and Western blot data showed that male tergal glands had significantly higher expression of Bla g 2 than the tissues of nymphs, a life stage not previously analyzed for its Bla g 2 content. Nymphal 7−8th tergites had significantly higher *Bla g 2* mRNA expression than nymphal heads in qPCR analysis. However, no difference between nymphal 7−8th tergites and other nymphal tissues was found during Western blot analysis. Although nymphs do not have tergal glands, it is likely that *Bla g 2* mRNA localizes or undergoes increased transcription in the cells of nymphal 7−8th tergites as they progress toward adulthood, particularly male nymphs. Tergites of last stage nymphs in *L. maderae* show localization of Lma‐p54 (an inactive aspartic protease) mRNA (Cornette et al., 2002). Given that male to female ratio in German cockroaches is ~1:1, it is likely that some of the sampled nymphs were males, which may have had tergal gland cells localized in the 7−8th tergite area and hence the higher expression of Bla g 2 in this tissue region.

In females (virgin), male tergal glands showed significantly higher levels of *Bla g 2* gene and protein expression compared to female head, wing, and gut tissues. The male tergal glands additionally had significantly higher Bla g 2 protein expression compared to the female carcass and 7−8th tergites. The female wings exhibited significantly higher Bla g 2‐Band 1 protein expression than the gut, which may possibly be due to the accumulation of environmental Bla g 2 on the wings as demonstrated by Arruda et al. (1995).

In gravid female tissues, there was no difference in *Bla g 2* gene expression among the head, wings, gut, carcass, ootheca, and male tergal gland tissues. However, male tergal glands exhibited significantly higher Bla g 2 protein expression in comparison to gravid female tissues, including the gut, wings, 7−8th tergites, carcass, and ootheca. This may be due to differences in quantification techniques between the Western blot and qPCR protocols. In this regard, melt curve data from qPCR experiments (data not shown) suggested amplification of a single *Bla g 2* band, whereas, in the Western blot experiments, two or three Bla g 2 protein bands were detected based on life stage likely due dimerization and posttranslational modifications as described later. Thus, separate quantification of Bla g 2 protein signals in multiple bands in Western blot experiments versus the collective analysis and quantification of *Bla g 2* mRNA in qPCR experiments could explain some of the observed differences.

The male tergal glands showed higher *Bla g 2* gene expression than the male head, gut, wings, and 5−6th tergites, as well as higher protein expression than the 5−6th tergites and carcass. The minor tissue‐specific differences in statistical significance between Bla g 2 protein and gene expression in male tissues may be due to the difference in quantification techniques between the Western blot protocol and the qPCR protocol, as mentioned in the case of the gravid females.

Proteomic approaches were further used to verify the identity of protein bands recognized by the Bla g 2 antiserum. The multiple Bla g 2 protein bands detected on the Western blots (i.e., two bands for nymphs, males, and females, and three bands for gravid females) that were ca. 35, 70, and 105 kDa in size were verified to contain peptides belonging to one or more Bla g 2 isoforms through peptide sequencing. Previous, antibody‐based detection and purification work from crude cockroach extracts estimated the size of the Bla g 2 allergen protein to be ca. 36 kDa (Pollart et al., 1991). Pollart et al. (1991) also reported detection of a dense protein band of ca. 70 kDa size in addition to the 36 kDa Bla g 2 protein band. Our Western blot study coupled with SDS‐PAGE and LC‐MS/MS‐based peptide sequencing shows that the 70 kDa band region also contains Bla g 2 peptides, thereby confirming the previous observation that partially purified cockroach extracts contain allergen or Bla g 2 proteins ranging from 25 to 70 kDa in size (Pollart et al., 1991; Schou et al., 1990; Twarog et al., 1977; Wu & Lan, 1988). More recent literature suggests the existence of a Bla g 2 dimer; Arruda et al. (1995) sequenced a 70 kDa protein, and the first 10 residues of this 70 kDa protein matched the NH_2_‐terminal sequence of Bla g 2, which indicated that the protein may be a Bla g 2 dimer. Furthermore, Pomés et al. (2007) and Li et al. (2008) confirmed that Bla g 2 dimerizes in a crystal complex with two Fab' molecules from the monoclonal antibody. Results of these previous studies, along with our peptide sequencing results, clearly show that Bla g 2 is forming a dimer that is complexed with our polyclonal antibody. The study by Li et al. (2008) also mentions that Bla g 2 dimers increase the IgE cross‐linking in mast cells that trigger the release of β‐hexosaminidase, meaning dimerization of Bla g 2 may increase its allergenicity.

The above evidence was not the only indication of multimerization or posttranslational modifications in Western blots. There was also a relatively high‐mass, ~95–105 kDa band detected in gravid female whole‐body samples in the Western blot experiments. This band with higher molecular weight was not discussed in any previous literature. After performing SDS‐PAGE and LC‐MS/MS sequencing on this 105 kDa band region from gravid females, we found that it contained Bla g 2 peptides. The low relative abundance of 0.083 for Bla g 2 proteins in the 105 kDa band could be due to the higher abundance of other proteins in the 105 kDa size range, including vitellogenin. However, none of the other major proteins detected in the 105 kDa band region had the epitope sequences necessary for the Bla g 2 antibody to bind to them. The consistency of this 105 kDa band in our gravid female whole‐body replicates and its absence in all other life stages indicate that the band is not due to contamination or un‐migrated 35 and 70 kDa proteins. It is also possible that Bla g 2 protein forms a trimer, either with other Bla g 2 isoforms or proteins that are not Bla g 2, or a mixture of both. Three 35 kDa Bla g 2 isoforms bound together would be 105 kDa in mass, as would different 35 or 70 kDa proteins that bind to Bla g 2 monomers. Alternatively, post‐translation modifications such as glycosylation of the dimeric form of the Bla g 2 protein (Do et al., 2017) could have increased its molecular weight by approximately 25–35 kDa, resulting in the high mass band of ~95–105 kDa molecular weight. Previously, the Lma‐p54 protein, which is homologous to Bla g 2, was predicted to have glycosylation sites (Cornette et al., 2002). The possibilities of Bla g 2 trimerization or posttranslational modifications need to be further examined using various empirical experimental approaches.

In summary, this in‐depth analysis of Bla g 2 expression in different tissues, sexes, and life stages has addressed some of the previous gaps in knowledge and provided us with important answers, including: (i) confirmation that *Bla g 2* mRNA is present in male tergal glands, (ii) male tergal glands being one of the tissues in which Bla g 2 protein is highly expressed, and (iii) existence of Bla g 2 dimers and potential multimers in various cockroach life stages and tissues. These results also allow us to develop pertinent questions for future research. For example, what Bla g 2 isoforms and associated proteins are present in whole bodies and tissues of *B. germanica*? What are the different functions of these proteins and how do they interact with one another? Do the Bla g 2 proteins that are present in tergal gland secretions function as a source of nutrition for females? Since Bla g 2 shares sequence similarity with pregnancy‐associated glycoproteins that are conducive to embryonic development in cows and other ungulates, does Bla g 2 play a role in the development of German cockroach embryos as well (Haugejorden et al., 2006; Wünschmann et al., 2005)? As demonstrated by Myers et al. (2018), RNAi can be used to assess the different functions of the different Bla g 2 isomers, multimers, and other proteins. Although there is scientific literature on suppression of the Bla g 2 protein by treating German cockroaches with ampicillin (Lee et al., 2021), there is currently no literature on gene expression silencing of *Bla g 2* and any resulting phenotypes. Additionally, co‐immunoprecipitation (Co‐IP) followed by LC‐MS/MS, are other valuable tools to identify and isolate the proteins that interact with Bla g 2.

## AUTHOR CONTRIBUTIONS

**Aaron Rodriques**: Conceptualization (equal); data curation (lead); formal analysis (lead); investigation (lead); methodology (equal); validation (lead); visualization (lead); writing—original draft (lead); writing—review and editing (equal). **Aaron J. Myers**: Conceptualization (equal); investigation (equal); methodology (equal). **Uma Aryal**: Data curation (equal); formal analysis (equal); investigation (equal); methodology (equal); supervision (equal); validation (equal); visualization (equal); writing—review and editing (equal). **Michael E. Scharf**: Conceptualization (supporting); funding acquisition (supporting); investigation (supporting); methodology (supporting); supervision (supporting); writing—review and editing (supporting). **Gary W. Bennett**: Funding acquisition (supporting); project administration (supporting); supervision (supporting); writing—review and editing (supporting). **Ameya Gondhalekar**: Conceptualization (lead); data curation (equal); funding acquisition (lead); methodology (equal); project administration (lead); supervision (lead); visualization (equal); writing—original draft (equal); writing—review and editing (equal).

## CONFLICTS OF INTEREST

The authors declare no conflicts of interest.

## Supporting information

Supporting information.
