# Supplementary material for: Expression profiles of an inactive aspartic protease (Bla g 2 allergen) in different tissues and developmental stages of the German cockroach (Blattella germanica)
Source: Arch Insect Biochem Physiol. 2022 Jun 1;111(2):e21918. doi: 10.1002/arch.21918 (PMC9541408; doi:10.1002/arch.21918)
Supplement: Supplementary file 1 — Supporting information. [file ARCH-111-e21918-s001.docx]

***Supplementary Information of***

**Expression profiles of an inactive aspartic protease (Bla g 2 allergen) in different tissues and developmental stages of the German cockroach (*Blattella germanica*)**

Aaron R. Rodriques^1^, Aaron J. Myers^1^, Michael E. Scharf^1,2^, Uma K. Aryal^3,4^, Gary W. Bennett^1^ and Ameya D. Gondhalekar^1^.

^1^Department of Entomology, Purdue University, West Lafayette, IN 47907, USA

^2^Entomology and Nematology Department, University of Florida, Gainesville, FL 32611

^3^Department of Comparative Pathobiology, Purdue University, West Lafayette, IN 47907, USA

^4^Purdue Proteomics Facility, Bindley Bioscience Center, Purdue University, West Lafayette, IN 47907, USA

******

**Figure S1.** A clustered column chart showing the Bla g 2 protein expression in various tissues [Head (H), Wing (W), Gut (G), 5^th^-6^th^ tergites (T5-T6), 7^th^-8^th^ tergites (T7-T8), Carcass (C), Ootheca (O) and Whole body (WB)] from various life stages [Nymphs (N), Gravid Females (GF), Virgin Females (VF), Males (M)] examined in this study. The relative intensity sum, is the sum of the relative intensities quantified in the Band 1, Band 2, and Band 3 regions of each tissue based on three biological replicates.
